# The Prevalence of Shoulder Pain and Its Functional Limitations Among Patients With Uncontrolled Diabetes

**DOI:** 10.7759/cureus.11487

**Published:** 2020-11-15

**Authors:** Abdulrahman Aljethaily, Abdulrahman Alshuwayrikh, Saleh Alkhonezan, Abdullah Alasmari, Mohammed Almakdob, Amjad Albogami, Abdulmalik Aloriney, Ibrahim Ahmed, Bader Alzahrani

**Affiliations:** 1 Medicine, College of Medicine, Imam Mouhammad Ibn Saud Islamic University, Riyadh, SAU; 2 Family Medicine, College of Medicine, Imam Mouhammad Ibn Saud Islamic University, Riyadh, SAU; 3 Family Medicine, Security Forces Hospital, Riyadh, SAU

**Keywords:** diabetes, shoulder pain, ases

## Abstract

Objectives

To investigate the prevalence of shoulder pain and its functional limitations among patients with uncontrolled diabetes mellitus (DM).

Methods

This is a cross-sectional study that was conducted over a period of four months from October 2019 to January 2020 and included all patients with uncontrolled DM (HbA1c > 9) who are visiting the diabetes clinic at Security Forces Hospital during the study period. Telephone interviews were held by a physician from the Family Medicine Department using a previously validated questionnaire, that is, the American Shoulder and Elbow Surgeons Evaluation Form.

Results

A total of 285 patients were included in the study; 156 (54.7%) were females and 129 (45.3%) were males. Most (51.1%) of the patients aged 45-64 years. The majority of the patients 58.9% had type II DM and 41.1% of them had type I DM. The mean HbA1c level was 10.56. Of the patients are having shoulder pain (109; 38.2%), 42.5% were between 45 and 64 years of age and 44.1% were between 65 and 96 of years. While 176 (61.8%) of the participants had no pain at all, 70.5% males and 54.5% females did not have shoulder pain (p<0.001). The mean shoulder pain intensity for all patients was 5.81(SD=3.21), ranging from 4.71 (SD=3.15) to 6.13 (SD=3.29), according to different age groups, and showed a significant correlation (p<0.05).

Conclusions

Increasing prevalence of shoulder dysfunction is making physicians alert regarding early diagnosis and management of the disease. Thus, it should be mandatory to include screening, prevention, and rehabilitation strategies for shoulder dysfunction in diabetic care programs to improve the daily lifestyle of the patients.

## Introduction

Diabetes mellitus (DM) is a chronic disease that has turned into a major risk to human health in recent years. It possesses a variety of complications such as nephropathy, retinopathy, heart disease, and stroke. Musculoskeletal disease is also one of the important symptoms observed in diabetic patients, which presents as Dupuytren's contracture, stiff hand syndrome, tendinitis, adhesive capsulitis (frozen shoulder), and periarthritis of the shoulder [1-2]. Among all these, shoulder pain is found to be the most common symptom, which is characterized by pain and restricted movement [3]. Shoulder pain can lead to hindrance in daily activities that can directly or indirectly influence the metabolic processes of our body, thus affecting the quality of life [4].

The prevalence of DM was observed to be around 151 to 171 million in the year 2000, and it is estimated that it will be three times by the year 2050 [5]. A study by Alqurashi et al [6] in 2011 on the population of Saudi Arabia revealed that the prevalence rate of diabetic patients was approximately 30%, with the involvement of 34.1% males and 27.6% females. Various studies reported that shoulder disorders are found to be more prevalent (27.5%) in diabetic patients than in patients suffering from general medical conditions (5%) [6-7]. The most common shoulder disorders observed in patients are frozen shoulder, also known as “adhesive capsulitis” and rotator cuff disease [6].

It has been observed that the underlying pathophysiological processes of shoulder pain involve capsular inflammation following fibrosis. This manifestation is modulated by various mediators such as growth factors, enzymes, inflammatory cytokines, and matrix metalloproteinases (MMPs) [8-9].

The function of the upper limb is primarily dependent on the shoulder joint. Past studies have shown a direct link between the duration of DM-associated shoulder pain and control of blood sugar levels. Studies have also observed the role of social, genetic, and environmental factors in this ailment [10-11]. Since years, shoulder involvement in diabetic patients is not very well understood [12]. In the population of Saudi Arabia, we observed that only a few studies [13-14] have been conducted that reveal the prevalence of chronic pain due to various musculoskeletal disorders in diabetic patients, but there was no study that observes the relationship of HbA1c with shoulder pain and the prevalence of shoulder pain in patients suffering from DM.

Thus, this study was conducted with the aim to determine the prevalence of shoulder pain and its functional limitations among patients with uncontrolled DM at Security Forces Hospital using the American Shoulder and Elbow Surgeons (ASES) Evaluation Form.

## Materials and methods

This is a quantitative cross-sectional study conducted over a period of four months from October 2019 to January 2020. We enrolled all patients with uncontrolled DM (HbA1c > 9) who visited the diabetes clinic at Security Forces Hospital over the study period by registering their telephone numbers. Also, we excluded all patients who had known to have psychiatric illness and thus incapable of answering questions, had gestational DM, were medically unstable, or were unwilling to participate. Then, telephone interviews were held by a physician from the Family Medicine Department using the ASES Evaluation Form.

The ASES Evaluation Form is developed by the Committee of American Shoulder and Elbow Surgeons to facilitate the assessment of shoulder problems. It is a scoring tool for shoulder pain and its functional limitations; 100 points are the maximum score, and half of the score (50%) is driven from the shoulder pain and its intensity and the other half is a cumulative score driven by 10 questions about daily activity. Each question can be scored as an ordinal scale (from 0 to 3 points). As the ASES scores higher, it correlates with better function and less pain.

The data were entered into a Microsoft Excel spreadsheet and analyzed using Statistical Package for the Social Sciences (SPSS) (IBM Corp., Armonk, NY, USA). All categorical variables, which include gender, marital status, smoking status, and educational level, were summarized and reported as frequency and percentage. Continuos variables were summarized as mean and standard deviation. p-Values of <0.05 were considered statistically significant.

This study was approved by the Ethics Committee of the Security Forces Hospital. Verbal consent was obtained from all participants, after having a brief explanation about the aims of the study and the contents of this telephone interview. The identity of the patients as well as the raw data, which include personal information, were kept confidential.

## Results

A total of 285 patients were included in the study. Most (51.1%) of the patients aged 45-64 years, whereas only 7% were younger than 18 years old. Of the patients, 156 (54.7%) were females and 129 (45.3%) were males, most (67.4%) of them were married, 16.5% were single, and only 1.8% were divorced. Most of the patients had a low level of education (79.3%), either secondary education or less. Also, 43.9% were unemployed, 9.8% were housewives, and only 3.2% were working in the private sector. Overall, 77.9% of the patients were never smokers and 12.3% were currently smoking. Type II D was prevalent in 58.9% and type I DM in 41.1%; the mean HbA1c level was 10.56. The duration of DM was more than 10 years in 82.1% of them, and 71.9% were on insulin injection treatment. Of the 285 patients, 109 (38.2%) had shoulder pain and the rest 176 (61.8%) had no pain at all. Concerning shoulder pain, 56.9% of patients between 45 and 64 years of age and 23.9% between 65 and 96 years stated that they have shoulder pain (p>0.05). Among males and females, 34.9% and 65.1%, respectively, had shoulder pain (p<0.001). Mostly, patients having more than secondary education did not have shoulder pain (p>0.05). The majority (75.2%) of participants who had shoulder pain never smoked (p<0.05). Of those having shoulder pain, the vast majority (61.5%) have type II DM, whereas 38.5% had type I DM. The participants who exercised regularly represented the majority (59.6%) of patients with shoulder pain (Table 1).

**Table 1:** Demographic characteristics of the study sample (n = 285). All variables were tested using Fisher's exact test. DM, diabetes mellitus; SD, standard deviation

Factor	All patients, n = 285 (100%)	Participants having shoulder pain, n = 109 (38.2%)	Participants not having shoulder pain, n = 176 (61.8%)	p-Value
Gender				
Male	129 (45.3%)	38 (34.9%)	91 (51.7%)	0.006
Female	156 (54.7%)	71 (65.1%)	85 (48.3%)	
Age, years				
0-17	20 (7.0%)	7 (6.4%)	13 (7.4%)	0.053
18-44	60 (21.1%)	14 (12.8%)	46 (26.1%)	
45-64	146 (51.2%)	62 (56.9%)	84 (47.7%)	
65-96	59 (20.7%)	26 (23.9%)	33 (18.8%)	
Occupation				
Unemployed	125 (43.9%)	66 (60.6%)	59 (33.5%)	0.000
House wife	28 (9.8%)	9 (8.3%)	19 (10.8%)	
Retired	27 (9.5%)	6 (5.5%)	21 (11.9%)	
Student	24 (8.4%)	3 (2.8%)	21 (11.9%)	
Civil servant	26 (9.1%)	11 (10.1%)	15 (8.5%)	
Private sector employee	9 (3.2%)	0 (0.0%)	9 (5.1%)	
Military personnel	46 (16.1%)	66 (12.8%)	32 (18.2%)	
Educational status				
Secondary education or lower	226 (79.3%)	93 (85.3%)	133 (75.6%)	0.124
Diploma	15 (5.3%)	4 (3.7%)	11 (6.3%)	
Bachelor	44 (15.4%)	12 (11.0%)	32 (18.2%)	
Master	0 (0%)	No valid cases		
Doctorate	0 (0%)			
Marital status				
Married	192 (67.4%)	75 (68.8%)	117 (66.5%)	0.000
Divorced	5 (1.8%)	2 (1.8%)	3 (1.7%)	
Widower	41 (14.4%)	26 (23.9%)	15 (8.5%)	
Single	47 (16.5%)	6 (5.5%)	41 (23.3%)	
Smoking status				
Not a smoker	222 (77.9%)	82 (75.2%)	140 (79.5%)	0.005
Former smoker	28 (9.8%)	18 (16.5%)	10 (5.7%)	
Currently smoking	35 (12.3%)	9 (8.3%)	26 (14.8%)	
Doing regular exercise				
No	165 (57.9%)	65 (59.6%)	100 (56.8%)	0.640
Yes	120 (42.1%)	44 (40.4%)	76 (43.2%)	
Type of DM				
DM type 1	117 (41.1%)	42 (38.5%)	75 (42.6%)	0.496
DM type 2	168 (58.9%)	67 (61.5%)	101 (57.4%)	
HbA1c, mean (SD)	10.56 (1.36)			
Duration of having DM				
Less than 1 year	10 (3.5%)	3 (2.8%)	7 (4.0%)	0.112
6-10 years	41 (14.4%)	10 (9.2%)	31 (17.6%)	
More than 10 years	234 (82.1%)	96 (88.1%)	138 (78.4%)	
Mode of current treatment				
Oral medications	80 (28.1%)	35 (32.1%)	45 (25.6%)	0.232
Insulin injections	205 (71.9%)	74 (67.9%)	131 (74.4%)	

The mean shoulder pain intensity for all patients was 2.22 (SD=3.45), ranging from 1.65 (SD=2.90) to 2.60 (SD=3.71), according to different age groups, and showed a significant correlation (p<0.05). And across gender, it was 1.46 (SD=2.61) in males 2.85 (SD=3.91) in females (p<0.001). Regarding different smoking status, the intensity of the pain ranged from 1.20 (SD=2.31) to 3.04 (SD=2.98), and regarding different durations of DM, it ranged from 1.20 (SD=1.98) to 2.39 (SD=3.52). The intensity was 1.93 (SD=3.25) in type I DM and 2.42 (SD=3.58) in type II DM (Table 2).

**Table 2:** Mean (SD) of shoulder pain intensity in demographic variables. DM, diabetes mellitus; SD, standard deviation

Variable	Mean shoulder pain intensity (SD)
All participants	2.22 (3.45)
Age, years	
0-17	1.65 (2.90)
18-44	1.13 (2.25)
45-64	2.60 (3.71)
65-96	2.58 (3.75)
p-Value	0.023
Gender	
Male	1.46 (2.61)
Female	2.85 (3.91)
p-Value	0.001
Smoking status	
Not a smoker	2.28 (3.62)
Former smoker	3.04 (2.98)
Currently smoking	1.20 (2.31)
p-Value	0.224
Duration of DM	
Less than 1 year	1.20 (1.98)
6-10 years	1.51 (3.21)
More than 10 years	2.39 (3.52)
p-Value	0.104
Type of DM	
DM type 1	1.93 (3.25)
DM type 2	2.42 (3.58)
p-Value	0.238

Table 3 demonstrates the participants’ answers to different shoulder pain-related problems. Regarding throwing a ball overhand, 10.1% of the patients could not do it, 36.7% had no problem doing it, and 26.6% had slight-to-moderate trouble in throwing a ball overhand. Also, 20.2% of the patients could not sleep over their shoulder, whereas 29.4% had no problem; 4.6% could not put on their coat without assistance, whereas 67.9% could. In addition, 4.6% of patients could not wash their back, whereas 63.3% had no difficulties in doing it. Regarding using toilet tissue, a very small percent (1.8%) could not use them without assistance, whereas 70.6% could. Also, 4.6% of the cases had no ability to comb or wash their hair, whereas 69.7% had no problem in doing it. In terms of weight lifting, 45.9% of the patients could not lift 10 kg above the level of their shoulder, whereas 17.4% could; 17.4% of the patients could not reach a shelf over their head, whereas 44% could. Due to shoulder pain and movement restriction, 25.7% of the cases could not perform their regular activities or do their full-time work, whereas 39.4% had no problem in doing their work or daily activities. Moreover, 29.4% could not play regular sports, whereas 34.9% could.

**Table 3:** Answers to shoulder pain.

Variable	No, n (%)	Moderate trouble, n (%)	Slight trouble, n (%)	Yes, n (%)
Can you throw a ball overhand?	11 (10.1%)	29 (26.6%)	29 (26.6%)	40 (36.7%)
Can you sleep on your shoulder comfortably?	22 (20.2%)	31 (28.4%)	24 (22%)	32 (29.4%)
Can you put on your coat unassisted?	5 (4.6%)	13 (11.9%)	17 (15.6%)	74 (67.9%)
Can you wash your back?	5 (4.6%)	20 (18.3%)	15 (13.8%)	69 (63.3%)
Can you use toilet tissue?	2 (1.8%)	14 (12.8%)	16 (14.7%)	77 (70.6%)
Can you comb/wash your hair?	5 (4.6%)	15 (13.8%)	13 (11.9%)	76 (69.7%)
Can you lift 10 kg above the level of your shoulder?	50 (45.9%)	28 (25.7%)	12 (11%)	19 (17.4%)
Can you reach a shelf over your head?	19 (17.4%)	24 (22%)	18 (16.5%)	48 (44%)
Does your shoulder allow you to work full time or perform regular activities?	28 (25.7%)	19 (17.4%)	19 (17.4%)	43 (39.4%)
Does your shoulder allow you to do your regular sports?	32 (29.4%)	23 (21.1%)	18 (14.7%)	38 (34.9%)

The mean shoulder ASES score for all patients was 82.18 (SD=27.20), ranging from 77.82 (SD=31.08) to 92 (SD=16.55), according to different age groups, with 87.88 (SD=21.71) in males and 77.46 (30.28) in females (p<0.001). It ranged from 75.83 (23.58) to 89.66 (SD=19.39) with different smoking status and from 80.54 (SD=28.12) to 90.66 (SD=15.53) in different durations of DM, with 83.93 (SD=25.87) in type I DM and 80.96 (SD=28.09) in type II DM. The mean shoulder ASES score for all patients showed significant association with age, gender, and duration of DM (p<0.05), whereas other demographic variables did not show any association (p>0.05) (Table 4).

**Table 4:** Mean (SD) ASES score in demographic variables. ASES, American Shoulder and Elbow Surgeons; DM, diabetes mellitus; SD, standard deviation

Variable	Mean of ASES score (SD)
All participants	82.18 (27.20)
Age, years	
0-17	86.0 (23.82)
18-44	92.0 (16.55)
45-64	79.38 (28.62)
65-96	77.82 (31.08)
p-Value	0.008
Gender	
Male	87.88 (21.71)
Female	77.46 (30.28)
p-Value	0.001
Smoking status	
Not a smoker	81.80 (28.47)
Former smoker	75.83 (23.58)
Currently smoking	89.66 (19.39)
p-Value	0.273
Duration of DM	
Less than 1 year	90.66 (15.53)
6-10 years	89.43 (22.48)
more than 10 years	80.54 (28.12)
p-Value	0.057
Type of DM	
DM type 1	83.93 (25.87)
DM type 2	80.96 (28.09)
p-Value	0.366

## Discussion

Musculoskeletal disease is one of the most common complications in DM patients. The global prevalence of DM among adults (over 18 years of age) has risen from 4.7% in 1980 to 9.5% in 2018 [15]. The shoulder or glenohumeral joint is a triaxial joint that connects the head of the humerus with the glenoid fossa of the scapula. This joint has greater mobility than any other joint in the body [16]. Among the various musculoskeletal diseases, shoulder pain is one of the most common complaints among DM patients. In general, it is characterized by pain and limited range of motion of one or both shoulders. Shoulder pain not only causes a decreased quality of life but also leads to disability in daily activities, and might interfere directly or indirectly with control of metabolic processes [17]. The exact mechanism that leads to the occurrence of shoulder pain in diabetic patients has not been recognized. The two conditions [7] that have similar mechanisms as of DM are (i) impairment of microcirculation and (ii) process of non-enzymatic glycosylation. It has been observed that hyperglycemia causes the development of non-enzymatic glycosylation products and leads to the formation of advanced glycosylation end-products (AGEs). AGEs escalate cross-linking in tendons, collagen, and ligaments, making them weaker and stiffer. They also relate with their receptors present on tenocytes and fibroblasts, thus causing inflammatory changes [18]. Moreover, the unfavorable microvascular situation created by hyperglycemia occurs around the shoulder joint as well. The impaired circulation causes overproduction of free radicals and tissue hypoxia causing potential apoptosis. This destruction causes damage to tissues of the joint and leads to various degenerative changes. The cross-linking collagen gathered in the shoulder capsule causes joint stiffness. Hyperglycemia leads to the chronic inflammatory process that increases the inflammation reaction in the synovium. All these factors lead to capsular fibrosis of the shoulder joint [19]. In Saudi Arabia, the prevalence and patterns of chronic pain due to musculoskeletal system among rheumatology patients were observed to be 42%. The most common sites were low back pain, neck pain, and shoulder pain, with a frequency of 52%, 41%, and 26%, respectively [20].

In this study, 38.2% of the diabetic patients suffered from shoulder pain, with 71% of them being females, especially in the age group of 45-64 years, whereas according to past studies [21-22], the shoulder pain prevalence in the general population was reported to be 2-5% only. Also, 68.8% of the married participants could be affected because of the increase in post-marriage stress levels compared to divorced, widower, or single.

Previously, studies have shown that the mode of anti-diabetic medications also affects the prevalence of shoulder pain. Non-insulin-dependent patients (type II) were 1.59 times more affected with shoulder pain than type I, and the results were comparable to past studies [23-24].

Cagliero et al. [19] found higher levels of HbA1c in diabetic patients with shoulder soft tissue musculoskeletal complaints. Arkkila et al. [11] also found that type 2 DM patients with inadequate glycemic control (HbA1c greater than 9%) had more shoulder capsulitis. Similar results were noted in our study, where the HbA1c levels were found to be 10.56±1.36, but no significant correlation between HbA1c and shoulder pain was found.

 Wong et al. [25] found that chronic illness, increasing age, marital discord, the presence of anxiety and depression, lack of exercise, and poor quality of life were found to be significantly associated with the presence of DM-associated shoulder pain. Similar results were noted in our study as 65% of patients were not doing regular exercise, and most of the patients were having a strenuous lifestyle because of the pressure of education. In our study, no association was found between smoking habit and prevalence (p=0.05) of shoulder pain in the patients. Regarding the overall effect of shoulder pain over the quality of daily life, it was noted that only 36.7% of patients did not have any trouble in throwing the ball overhand. In addition, 29.4% of the patients replied that they could sleep on their affected shoulder comfortably, and 67.9%% of the patients could wear their coat unassisted. When asked about washing back while bathing and using toilet tissue paper, 63.3% and 70.6% of patients, respectively, replied positively to it. Also, 69.7% of the patients with DM-associated shoulder problem replied that they could do their hairwashing and comb independently, and 45.9% of patients totally refused to lift 10 kg of weight above their shoulder because of unbearable shoulder pain and decreased mobility of their affected shoulder during this activity. Only 44% of the patients were able to reach a shelf above their heads easily. The majority (60.6%) of patients felt unconformable to work full time or perform regular activities with DM-associated shoulder pain, and 65.1% of patients responded that they could do their regular sports activities. All these responses showed that chronic shoulder pain put a substantial risk on health outcomes such as physical activity and dietary habits, and these lifestyle changes may, in turn, aggravate type 2 DM in these patients. The results were in agreement with many past studies [26].

The main limitation of the study is that due to the small sample size, no significant association between the results and the duration of DM was found. Moreover, it can be argued that the questions on smoking could be subjected to reporting bias. Due to its cross-sectional design, this study does not allow an analysis of prior glycemic control in the occurrence of pain and shoulder dysfunction.

We encourage that future interventional trials or prospective cohort studies be conducted in order to verify the causality for the outcomes of this study, which could help in the development of new strategies to minimize the suffering of diabetic individuals and promote a good quality of life. This study is recent and addresses the current gap in the contemporary literature.

## Conclusions

A high prevalence of shoulder dysfunction in diabetic individuals will urge clinical practitioners to examine and identify musculoskeletal findings in diabetic patients. Early diagnosis and treatment of DM can reduce the development of shoulder pain that can cause impairment in the routine functioning of diabetic patients. Early identification of signs of musculoskeletal complications can prove to be a valuable finding in the comprehensive care of diabetic patients. The increasing prevalence of shoulder dysfunction is making physicians alert regarding early diagnosis and management of the disease. Thus, it should be mandatory to include screening, prevention, and rehabilitation strategies for shoulder dysfunction in diabetic care programs to improve the daily lifestyle of the patients.

## References

[1] Lebiedz-Odrobina D, Kay J (2010). Rheumatic manifestations of diabetes mellitus. Rheum Dis Clin North Am.

[2] Arkkila PE, Gautier JF (2003). Musculoskeletal disorders in diabetes mellitus: an update. Best Pract Res Clin Rheumatol.

[3] Garcilazo C, Cavallasca JA, Musuruana JL (2010). Shoulder manifestations of diabetes mellitus. Curr Diabetes Rev.

[4] Lindgren I, Gard G, Brogårdh C (2018). Shoulder pain after stroke - experiences, consequences in daily life and effects of interventions: a qualitative study. Disabil Rehabil.

[5] Manninen P, Riihimäki H, Heliövaara M, Mäkelä P (1996). Overweight, gender and knee osteoarthritis. Int J Obes Relat Metab Disord.

[6] Alqurashi KA, Aljabri KS, Bokhari SA (2011). Prevalence of diabetes mellitus in a Saudi community. Ann Saudi Med.

[7] Thomas SJ, McDougall C, Brown ID (2007). Prevalence of symptoms and signs of shoulder problems in people with diabetes mellitus. J Shoulder Elbow Surg.

[8] Cho CH, Song KS, Kim BS, Kim DH, Lho YM (2018). Biological aspect of pathophysiology for frozen shoulder. Biomed Res Int.

[9] Wyatt LH, Ferrance RJ (2006). The musculoskeletal effects of diabetes mellitus. J Can Chiropr Assoc.

[10] Laslett LL, Burnet SP, Redmond CL, McNeil JD (2008). Predictors of shoulder pain and shoulder disability after one year in diabetic outpatients. Rheumatology (Oxford).

[11] Arkkila PE, Kantola IM, Viikari JS, Ronnemaa T Shoulder capsulitis in type I and II diabetic patients: association with diabetic complications and related diseases. Ann Rheum Dis.

[12] Abate M, Schiavone C, Salini V, Andia I (2013). Management of limited joint mobility in diabetic patients. Diabetes Metab Syndr Obes.

[13] El-Metwally A, Shaikh Q, Aldiab A (2019). The prevalence of chronic pain and its associated factors among Saudi Al-Kharj population; a cross sectional study. BMC Musculoskelet Disord.

[14] Aldossari KK, Aldiab A, Al-Zahrani JM (2018). Prevalence of prediabetes, diabetes, and its associated risk factors among males in Saudi Arabia: a population-based survey. J Diabetes Res.

[15] Sarwar N, Gao P, Seshasai SR (2010). Diabetes mellitus, fasting blood glucose concentration, and risk of vascular disease: a collaborative meta-analysis of 102 prospective studies. Lancet.

[16] Kisner C, Colby LA, Borstad J (2018). The shoulder and shoulder girdle. Therapeutic Exercise: Foundations and Techniques.

[17] Lan Hsu C, Sheu WHH (2016). Diabetes and shoulder disorders. J Diabetes Investig.

[18] Goldin A, Beckman JA, Schmidt AM, Creager MA (2006). Advanced glycation end products: sparking the development of diabetic vascular injury. Circulation.

[19] Cagliero E, Apruzzese W, Perlmutter G, Nathan DM (2002). Musculoskeletal disorders of the hand and shoulder in patients with diabetes mellitus. Am J Med.

[20] Moussa S, Al Zaylai F, Alomar A, Al Oufi H, AL Nodali N, Alshmmry RTA, Al-Enzy S (2015). Musculoskeletal pain in hail community: medical and epidemiology study; Saudi Arabia. Int J Sci Res.

[21] Moren-Hybbinette I, Moritz U, Schersten B The clinical picture of the painful diabetic shoulder--natural history, social consequences, and analysis of concomitant hand syndrome. Acta Med Scand.

[22] Lequesne M, Dang N, Bensasson M, Mery C (1977). Increased association of diabetes mellitus with capsulitis of the shoulder and shoulder-hand syndrome. Scand J Rheumatol.

[23] Zreik NH, Malik RA, Charalambous CP (2016). Adhesive capsulitis of the shoulder and diabetes: a meta-analysis of prevalence. Muscles Ligaments Tendons J.

[24] Yian EH, Contreras R, Sodl JF (2012). Effects of glycemic control on prevalence of diabetic frozen shoulder. J Bone Joint Surg Am.

[25] Wong WS, Fielding R (2011). Prevalence and characteristics of chronic pain in the general population of Hong Kong. J Pain.

[26] Lin TT, Lin CH, Chang CL, Chi CH, Chang ST, Sheu WH (2015). The effect of diabetes, hyperlipidemia, and statins on the development of rotator cuff disease: a nationwide, 11-year, longitudinal, population-based follow-up study. Am J Sports Med.

